# Evidence gaps in the effects of exercise on SASP-Related biomarkers in older adults: a systematic review and meta-analysis of randomized controlled trials

**DOI:** 10.1186/s12877-026-07025-5

**Published:** 2026-02-06

**Authors:** Eleuterio A. Sánchez-Romero, Oliver Martínez-Pozas, Samuel Fernández-Carnero, Álvaro Romero-Rosado, Rob Sillevis, Juan Nicolás Cuenca-Zaldívar

**Affiliations:** 1 Research Group in Nursing and Health Care, Puerta de Hierro Health Research Institute-Segovia de Arana (IDIPHISA), Majadahonda, 28222 Spain; 2 Department of Rehabilitation Sciences, Florida Gulf Coast University, Fort Myers, FL 33965 USA; 3 Interdisciplinary Research Group on Musculoskeletal Disorders, Madrid, 28014 Spain; 4 Physiotherapy and Orofacial Pain Working Group, Sociedad Española de Disfunción Craneomandibular y Dolor Orofacial (SEDCYDO), Madrid, 28009 Spain; 5 Departamento de Fisioterapia, Universidad de Alcalá, Facultad de Enfermería y Fisioterapia, Alcalá de Henares, 28801 Spain; 6 Physical Therapy Unit, Primary Health Care Center “El Abajón”, Las Rozas de Madrid, 28231 Spain

**Keywords:** Cellular senescence, Physical exercise, Aging, Senescence-Associated secretory phenotype (SASP), Inflammatory biomarkers, p16^INK4a^, p21, Senescence markers, Inflammaging

## Abstract

**Background:**

Cellular senescence is a hallmark of aging, characterized by the secretion of proinflammatory factors known as the senescence-associated secretory phenotype (SASP). While physical exercise is proposed as a potential modulator of cellular aging, its effect on specific senescence biomarkers in older adults remains unclear.

**Methods:**

We conducted a systematic review and meta-analysis following PRISMA guidelines (PROSPERO: CRD42024623676). Randomized controlled trials (RCTs) involving participants aged ≥ 60 years were included if they compared structured exercise interventions with control conditions and reported biomarkers of cellular senescence. A Bayesian meta-analysis was performed, and the risk of bias was assessed using the Cochrane RoB 2.0 tool.

**Results:**

Three RCTs (*n* = 1447; age range 66–79; 64.2% female) met the inclusion criteria. Two were eligible for meta-analysis, focusing on SASP-related cytokines (CCL2, IL-6, IL-8, TNF-α). No statistically significant differences were observed between the exercise and control groups. One excluded study reported increased SIRT levels following resistance training, but these are not standard senescence markers. Notably, none of the included RCTs evaluated key upstream markers such as p16^INK4a^ or p21, highlighting a critical gap in current research.

**Conclusions:**

This review found no conclusive evidence that exercise training affects canonical markers of cellular senescence in older adults. The biomarkers analyzed were limited to SASP-related cytokines, which reflect downstream inflammation rather than primary senescence regulation. Future studies should use standardized protocols to evaluate upstream markers such as p16^INK4a^ and p21 to better understand the impact of physical activity on cellular aging.

**Supplementary Information:**

The online version contains supplementary material available at 10.1186/s12877-026-07025-5.

## Introduction

Aging is a complex and multifactorial biological process involving progressive decline in physiological function, increased vulnerability to disease, and altered homeostasis [1]. Among its various hallmarks, the accumulation of senescent cells contributes to tissue dysfunction and development of age-related conditions [1, 2]. Senescence is typically driven by replicative stress, DNA damage, inflammation, and metabolic dysfunction [2], and is characterized by stable cell cycle arrest and the acquisition of a pro-inflammatory profile known as the senescence-associated secretory phenotype (SASP) [3].

Although the effects of exercise on systemic inflammation and SASP have been extensively reviewed [4, 5], little is known about its effect on upstream regulators of cellular senescence, such as p16^INK4a^ and p21, two cyclin-dependent kinase inhibitors associated with irreversible cell cycle arrest [1, 6]. While SASP cytokines are frequently measured as downstream indicators of senescence, they do not capture upstream regulators, such as p16^INK4a^ and p21, which mediate irreversible cell cycle arrest and represent more direct markers of senescence. These markers represent core mediators of senescence, are independent of inflammation, and have shown predictive value for functional decline and age-related outcomes in humans [1].

Recent advances suggest that exercise may directly modulate senescence pathways, lowering circulating levels of p16^INK4a^ and p21 in peripheral blood T cells and muscle tissue [1, 7, 8]. Moreover, evidence from structured training programs indicates that reductions in these markers are correlated with improvements in physical performance, thus supporting their utility as therapeutic targets and clinical biomarkers [1, 6].

Bautmans et al. (2021) [4] conducted a comprehensive review of the anti-inflammatory effects of exercise in older adults, focusing primarily on cytokines and CRP. However, their analysis did not include cellular senescence markers beyond SASP, such as p16^INK4a^ or p21, nor did they systematically evaluate trials targeting these outcomes. Hence, a clear gap remains regarding the effect of physical exercise on primary senescence markers in aging populations [9].

This systematic review and meta-analysis of randomized controlled trials (RCTs) aimed to address this gap by evaluating the effects of exercise on the biomarkers associated with cellular senescence. Although we initially sought to assess upstream markers, such as p16^INK4a^ and p21, none of the included RCTs reported data on these regulators. Therefore, our study focused on SASP-related cytokines that are commonly used as indirect indicators of senescence. By highlighting the lack of randomized trials assessing upstream senescence regulators, this study contributes to the growing field of geroscience by identifying critical gaps and emphasizing the need for targeted biomarker research. We acknowledge that SASP-related cytokines represent only one facet of the complex senescence phenotype and are insufficient to fully define cellular senescence in the absence of upstream canonical markers. SASP includes inflammatory cytokines like IL-6 and TNF-α, which are frequently used in clinical trials as surrogate indicators of senescence-related processes, but also reflect generalized inflammaging.

## Methods

This systematic review and meta-analysis followed the Preferred Reporting Items for Systematic Reviews and Meta-Analyses (PRISMA) guidelines [10] and was registered in PROSPERO under the identifier CRD42024623676.

### Search strategy

PubMed, EMBASE, Cochrane, Web of Science (WoS), and Scopus databases were searched for publications up to January 2025 without date restrictions [11]. The reference lists of the selected studies were scrutinized to identify additional relevant sources.

The search strategy employed a combination of medical subject headings (MeSH terms) and non-MeSH terms using Boolean operators (OR and/or AND) to connect them. Examples of MeSH terms used included “Exercise,” “Cellular Senescence,” and “Aging,” while non-MeSH terms such as “Physical Activity,” Senescence Markers,” and “Older Adults” were also included. The complete search strategy, including the PubMed search protocol (adapted for other databases as needed), is available in Appendix A.

Two independent reviewers (E.A.S.R., O.M.P) conducted the search independently using identical methodologies, and any disagreements were resolved through consensus.

### Eligibility criteria

The eligibility criteria (following the PICO strategy [12]) were as follows: research participants consisted of older adults (aged 60 years and above) without restrictions on sex or ethnicity. The intervention and comparison encompassed structured exercise programs, including strength training, endurance training, and combined exercise interventions, compared with usual care controls or controls undergoing alternative interventions. The outcomes focused on biomarkers of cellular senescence (e.g., SASP). Only RCT were included in this study.

In all included RCTs, senescence-related biomarkers were assessed in blood-derived samples (serum or plasma) but not in tissue biopsies or cellular lysates.

### Procedures

Two researchers (E.A.S.R., O.M.P) independently screened the titles and abstracts. Any conflicts among raters were settled through consensus or when necessary, a third rater settled the conflict. Data extraction was performed by two researchers (E.A.S.R., O.M.P.) based on the author(s) and publication year, study objectives, research design, population, intervention, outcome measurements, and reported findings. This study was developed in accordance with the Cochrane Handbook for Systematic Reviews of Interventions [13].

### Risk of bias assessment

The risk of bias in the included studies was evaluated by two independent reviewers (E.A.S.R. and O. M. P.), and any disagreements were resolved through consensus or mediation by a third reviewer (S.F.C.).

For randomized controlled trials (RCTs), the “Revised Cochrane Risk of Bias Tool for Randomized Controlled Trials (RoB 2.0)” was utilized to examine five areas: bias from the randomization process, deviations from intended interventions, missing outcome data, outcome measurement, and selection of reported results [14]. Each area was rated as “low risk,” “some concerns,” or “high risk,” with the overall study classification following the same categories [14]. Inter-rater reliability was estimated using the kappa coefficient (κ), where κ > 0.7 indicates a high level of agreement between the reviewers; κ of 0.5–0.7, a moderate level of agreement; and κ < 0.5 a low level of agreement [15].

### Data analysis

For statistical analysis, the R Ver 4.1.3 program (R Foundation for Statistical Computing, Institute for Statistics and Mathematics, Welthandelsplatz 1, 1020 Vienna, Austria) and the *bayesmeta* [16] package were used.

For studies such as Fielding et al. [17], in which data were reported using the median and median absolute deviation (MAD), the data were assumed to follow a normal distribution, based on the description of the methodology provided by the authors. Therefore, the median was used as a proxy for the mean, and the standard deviation was derived from the MAD using validated formulas [18, 19].

In case of a limited number of eligible studies (*n* < 10), we performed a Bayesian meta-analysis using a random-effects model [20, 21], with the pre-post intervention difference and the mean difference (MD) between groups as the effect size. When not directly reported, these values were estimated using the standard Cochrane-recommended formulas [22], assuming a conservative pre-post correlation coefficient of 0.7 [23], as commonly adopted in other meta-analyses [24–27].

The selection of the prior parameters for the models followed the method proposed by Ott et al. [28], comparing weakly informative priors with two reference posterior distributions (non-informative Jeffreys and Half-normal priors), and selecting the distribution that yielded greater precision and informativeness.

Finally, publication bias was assessed using the D measure from the Copas Bayesian robust selection model, interpreted as negligible (< 0.25), moderate (0.25–0.5), high (0.5–0.75), or very high (> 0.75) [29].

## Results

**Fig. 1:**
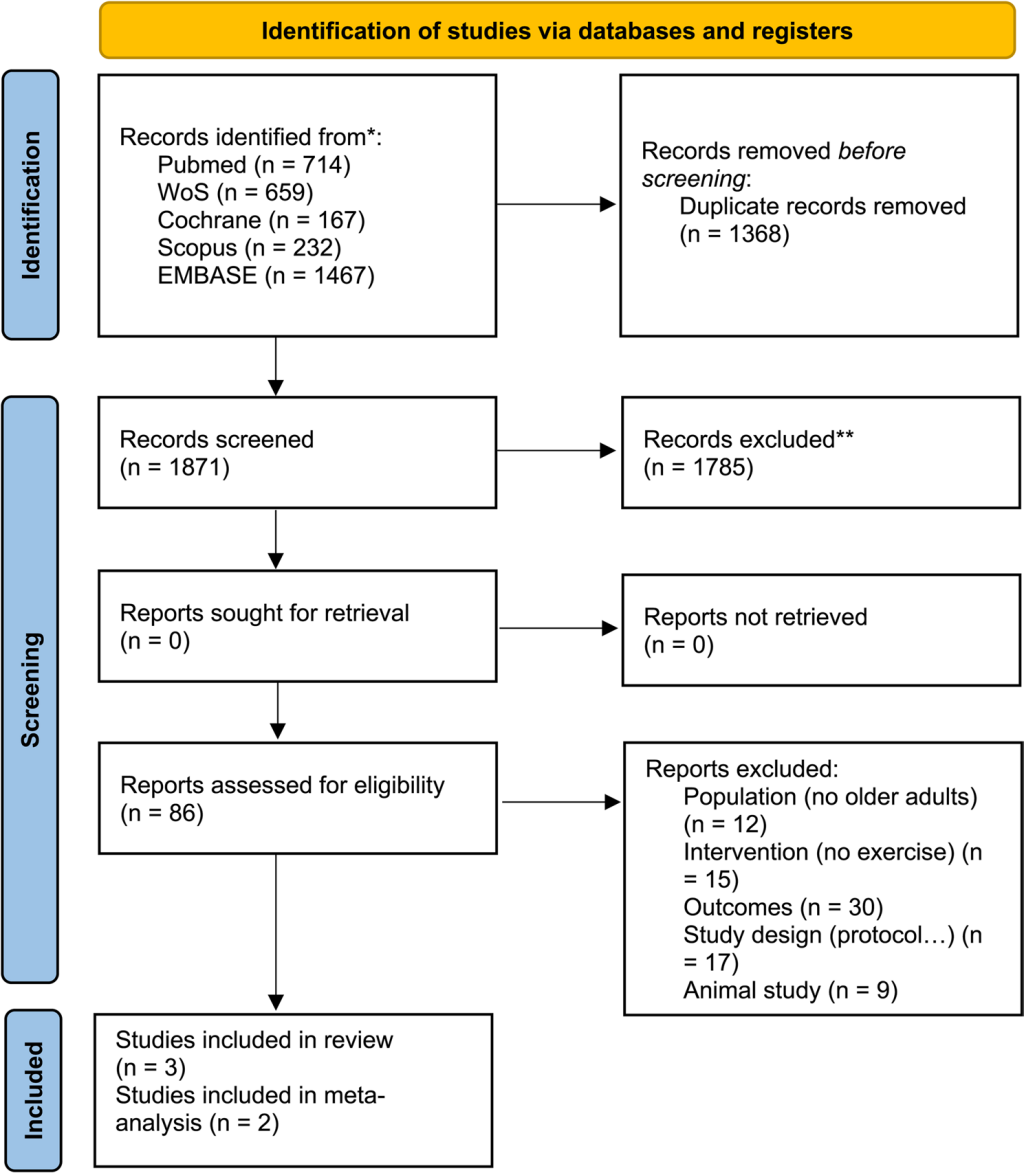
PRISMA 2020 flow chart

The database search yielded 3239 articles. After removing duplicates and screening the titles and abstracts, 86 articles were assessed for eligibility. After excluding the population (*n* = 12, no older adults), intervention (*n* = 15, no physical activity), outcomes (*n* = 30, no targeted outcomes), study design (*n* = 17, including protocols, systematic reviews, etc.), and species (*n* = 9, animals), a total of three randomized controlled trials were included in the present study [17, 30, 31]. A flowchart of the selection process and reasons for exclusion is shown in Fig. 1.

### Characteristics of the studies

Three studies encompassing 1447 older adults were included in this review. Among the participants, 930 were female (64.2%) and 517 were male (35.8%), with ages ranging from 66 to 79 years. In the intervention group, all studies implemented resistance training, while two also incorporated aerobic training [17, 30], with the duration of the interventions being between six weeks and three years. The control group in all studies received standard care, which consisted of health education and maintenance of participants’ usual lifestyle. Various SASP-related measures have been evaluated in several studies. Although the qualitative analysis addressed all SASP-related outcomes examined, a meta-analysis was conducted solely for outcomes that appeared in at least two studies, specifically CCL2, IL-6, IL-8, and TNFα. The additional details of the included studies are presented in Table 1.

**Table 1:** Summary of included studies and senescence-related biomarkers

Author (year)	Study design	Population	Sample size	Intervention	Control group	Outcomes	Results
Despeghel (2021) [30]	RCT	Older physically inactive adults	*n*=40 (int: 46.6%m, mean age: 70.4 ± 5.3 y/o) (con: 70%m, mean age: 69.8 ± 4.4 y/o)	*n*=30 Strength and aerobic training, 2 times per week for 6 weeks	*n*=10 Usual care (original lifestyle)	Different SASP-related outcomes (CCL2, CXCL13, ICAM1, IL1α, IL1RA, IL2, IL6, IL8, IL10, IL18, TNFα, VEGF)	No statistically significant differences were observed among the groups for any of the outcomes assessed. Within-group analyses indicated that the intervention group experienced significant reductions in IL-2, IL-6, IL-8, IL-10 and VEGF following the intervention, whereas the control group demonstrated significant decreases solely in IL-2.
Fielding (2024) [17]	RCT	Sedentary older adults (<150 min/week moderate PA)	*n*=1377 (int: 65%f, mean age: 78.6 ± 5.23 y/o) (con: 66%f, mean age:79.1± 5.20 y/o)	*n*=684 2 times per week supervised aerobic and resistance exercise. Established 150 min/week of PA at moderate intensity.	*n*=693 Usual care (education about healthy aging)	Different SASP-related outcomes (ACTIVIN A, ADAMTS13, EOTAXIN, FAS, GDF15, ICAM1, IL6, IL7, IL8, IL15, MCP1, MDC, MMP1, MMP2, MMP7, MMP9, MPO, OPN, PAI1, PARC, RAGE, RANTES, SOST, TNFα, TNFR1, TNFR2, VEGF-A)	No statistically significant within-group (pre–post) or between-group changes were observed in any of the SASP outcomes
Hoosmand-Moghadam (2020) [31]	RCT	Older adults physically active	*n*=30 (100%m, mean age: 66.2 ± 0.57 y/o)	*n*=15 12 weeks of resistance training, 3 times per week.	*n*=15 Usual care (original lifestyle)	SIRT1, SIRT3, SIRT6, PGC-1α	The intervention group exhibited statistically significant increases in SIRT levels, whereas the control group showed no significant changes. Additionally, there were no significant within-group changes observed in PGC-1α for either group.

*Abbreviatures*: *RCT* Randomized Controlled Trial, *int* intervention, *con* control, *SASP* Senescence-Associated Secretory Phenotype, *PA* Physical Activity, *f* female, *m* male

Table 1. Summary of Included Randomized Controlled Trials and Senescence-Related Biomarkers Assessed.

All biomarkers were quantified using blood samples. None of the included trials employed tissue-derived or cell-specific lysates to assess senescence markers such as p16^INK4a^ or p21.

### Risk of bias

**Fig. 2:**
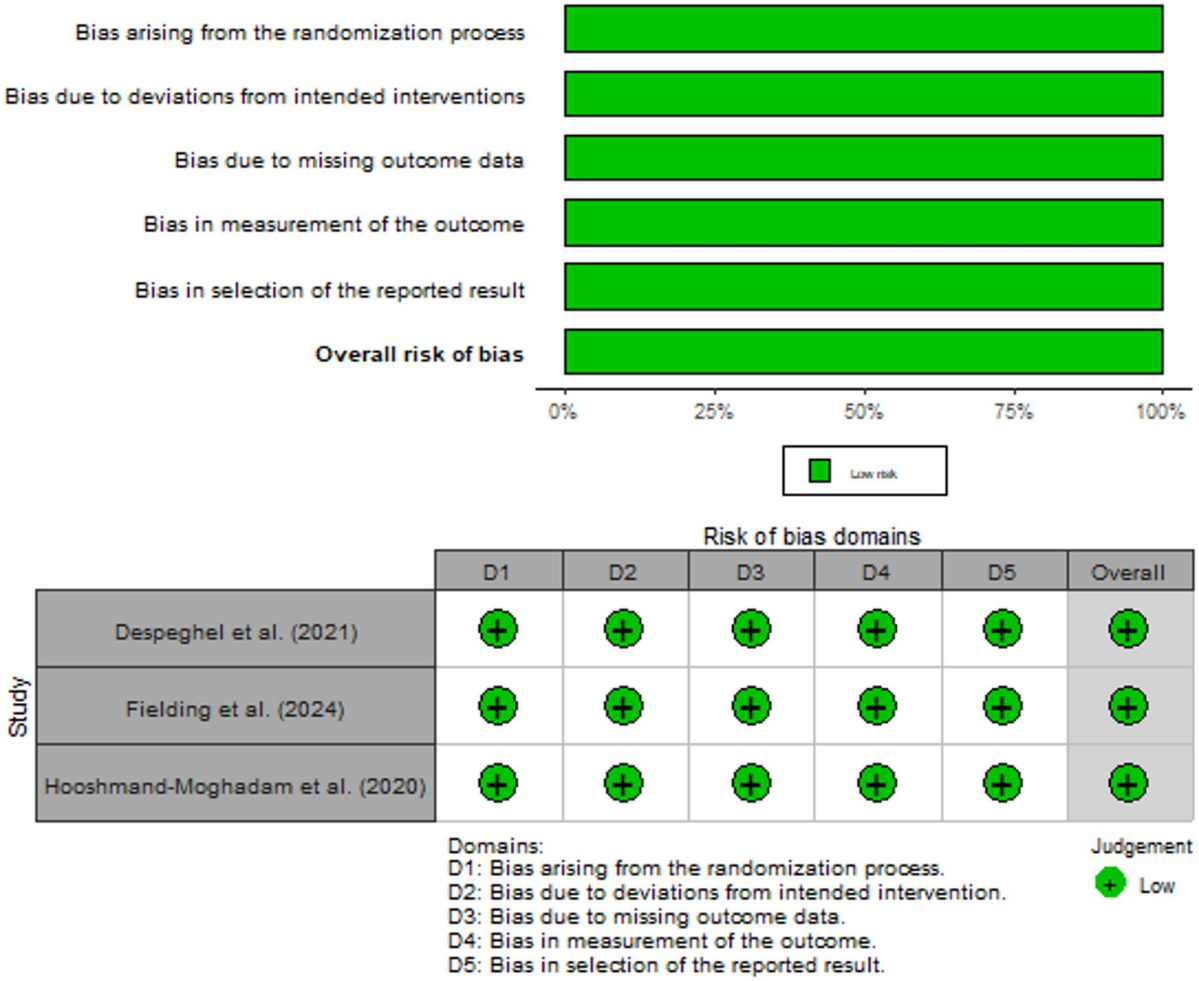
Risk of bias of randomized controlled trials (RoB 2.0)

All the included studies were identified as having a low risk of bias [17, 30, 31]. Figure 2 illustrates the risk of bias associated with the included studies.

### Synthesis of results

Three studies were included in this review [17, 30, 31]. However, one study was excluded from the meta-analysis because its outcome measurements did not encompass biomarkers directly related to SASP or core senescence markers (e.g., p16^INK4a^, p21) but instead focused on SIRT proteins, which are involved in aging regulation and oxidative stress pathways [31]. While the findings from this study suggest that resistance training may increase SIRT levels compared to the control, supporting its potential benefit in aging-related processes, these outcomes were not comparable with those of the other included trials and thus were not eligible for quantitative synthesis. Additionally, the small sample size and male-only sample limited the generalizability of the findings.

In a meta-analysis of two studies [17, 30], for all variables, the width of the confidence intervals was slightly higher in the Berger-Deely distribution (Supplementary Material, Table 1). However, the prior distribution parameters indicated that the Berger-Deely model was more informative, exhibiting reduced signed informativeness and a Hellinger distance closer to the anti-conservative Jeffreys distribution than to the conservative half-normal. Therefore, the Berger-Deely distribution was selected, except for biomarker CCL2, where the DuMouchel distribution presented a better balance between interval width and informativeness (Supplementary Material, Table 1, and Fig. 1).

**Fig. 3:**
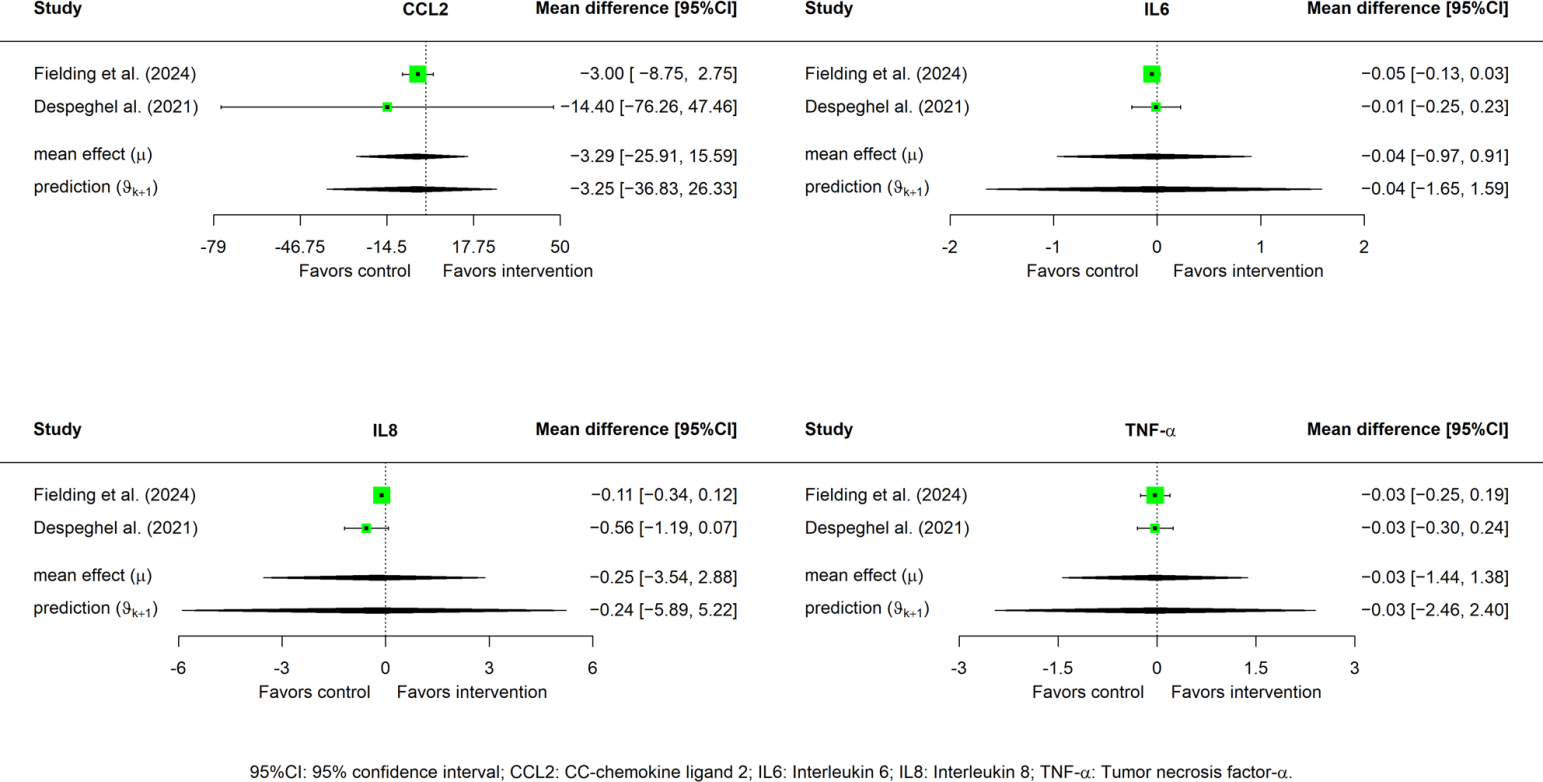
Outcome forest plots

There were no statistically significant differences between the exercise and control groups in CCL2 [MD = -3.639, 95% CI (-25.91, 15.594); τ = 3.207, 95% CI (0.092, 39.819] ), IL-6 [MD = -0.036, 95% CI (-0.968, 0.908); τ = 0.142, 95% CI (0.017, 3.288] ), IL-8 [MD = -0.284, 95% CI (-3.543, 2.877); τ = 0.507, 95% CI (0.062, 10.987] ), and TNF-α [MD = -0.03, 95% CI (-1.437, 1.377); τ = 0.219, 95% CI (0.029, 4.906] ), although trends suggested a greater reduction in biomarkers in the exercise group (Fig. 3).

Across all models, the posterior distribution of the effect size remained centered around zero, even as heterogeneity increased (Supplementary Material, Fig. 2A). The posterior distribution of the overall effect followed a normal distribution (Supplementary Material, Fig. 2B), whereas the heterogeneity parameter displayed a right-skewed non-normal distribution (Supplementary Material, Fig. 2C). Moreover, both the posterior distributions of the effect and predictive distributions were normal and closely aligned (Supplementary Material, Fig. 3). All models presented negligible publication bias (D = 0.206 for CCL2, 0.18 for IL6, 0.145 for IL8, and 0.218 for TNF-α).

### Certainty of evidence (GRADE Assessment)

We assessed the certainty of the evidence using the GRADE framework for the four SASP-related cytokines included in the meta-analysis. As shown in Table 2, the overall certainty was rated as very low for all outcomes, mainly because of serious imprecision (wide confidence intervals) and inconsistency (moderate to high heterogeneity).

**Table 2:** GRADE summary of findings: certainty of evidence for SASP-Related biomarkers

Biomarker	No. of studies	Participants (total)	Effect (MD [95% CI])	Risk of Bias	Inconsistency	Indirectness	Imprecision	Publication Bias	Overall Certainty
IL-6	2	210	-0.036 [-0.968, 0.908]	Low	Low–Moderate	No	Serious	None	⬤◯◯◯ Very low
TNF-α	2	210	-0.030 [-1.437, 1.377]	Low	Moderate	No	Serious	None	⬤◯◯◯ Very low
IL-8	2	210	-0.284 [-3.543, 2.877]	Low	Serious	No	Very serious	None	⬤◯◯◯ Very low
CCL2 (MCP-1)	2	210	-3.639 [-25.91, 15.594]	Low	Serious	No	Very serious	None	⬤◯◯◯ Very low

GRADE Working Group classification: ⬤⬤⬤⬤ High; ⬤⬤⬤◯ Moderate; ⬤⬤◯◯ Low; ⬤◯◯◯ Very low. Certainty downgraded based on risk of imprecision (wide confidence intervals) and inconsistency (heterogeneity among effect sizes). No concerns regarding risk of bias or publication bias were identified

The number of included studies was small, and no significant publication or risk of bias was identified. These findings reinforce the need for high-quality RCTs with standardized outcomes and sufficient power to improve the certainty of evidence.

## Discussion

Despite the growing evidence that exercise can modulate cellular aging, the current literature has several important limitations that temper firm conclusions. Small sample sizes are a common issue, and many interventional studies have been preliminary trials or sub-studies with only a few dozens of participants. This lack of statistical power can lead to inconclusive results, and may not capture the variability in responses among the broader older adult population. This systematic review and meta-analysis evaluated the effectiveness of physical exercise in modulating the biomarkers of cellular senescence in older adults. Only three studies were analyzed based on the inclusion criteria, and two were included in the meta-analysis. No statistically significant differences were found in the levels of the biomarkers CCL2, IL-6, IL-8, or TNF-α either during the intervention period or when comparing pre- and post-intervention values. These results suggest that physical exercise has little impact on the specific SASP-related cytokines assessed, which only partially reflect the cellular senescence process. However, it is important to acknowledge that these biomarkers are limited to SASP-related cytokines, whichalthough relevant, do not fully reflect the molecular complexity of cellular senescence. In particular, no studies have assessed upstream senescence regulators, such as p16^INK4a^ or p21, despite their relevance. This discrepancy between the intended scope and available evidence reflects a broader gap in the field and highlights the need for future trials to explicitly target these endpoints.

One study [31] reported an increase in SIRT levels following resistance training. Although SIRT proteins are implicated in aging-related pathways, they are not considered standard markers of cellular senescence. Therefore, SIRT was not included in this meta-analysis. This distinction is crucial to ensure methodological consistency and scientific precision in interpreting senescence outcomes.

Exercise modulates the inflammatory milieu and senescence-associated secretory phenotypes (SASP). Aging is characterized by sterile low-grade inflammation, partly fueled by senescent cells that secrete pro-inflammatory cytokines and chemokines. Previous studies have shown that exercise can reduce the accumulation of senescent cells in tissues and modulate components of the senescence-associated secretory phenotype (SASP) [32–36]. Nevertheless, these studies were not included in our review because they did not meet the eligibility criteria, which required a randomized controlled trial (RCT) design, specific biomarker reporting, and older adult populations. Our aim was to conduct rigorous synthesis based on high-level evidence.

An observational study by Fielding et al. [32] identified associations between high levels of SASP-related biomarkers and impaired physical function. While this supports the clinical relevance of senescence biomarkers, the difference in design compared to interventional trials limits direct comparability. Moreover, the absence of significant findings in our meta-analysis may reflect the small number of included studies, variability in intervention duration, and differences in the types of exercise program implemented.

Although these mechanisms were not investigated in the RCTs included in our review, preclinical studies suggest that exercise may influence senescence pathways via activation of AMPK and SIRT1, enhancement of mitochondrial function, and improved antioxidant capacity [34–36]. These hypotheses are cited here to provide a broader biological context, but they should be interpreted with caution as they are not directly supported by our findings. Moreover, we recognize that a comprehensive assessment of senescence requires integration of multiple markers (e.g., p16^INK4a^, p21, SA-β-gal, telomere-associated foci), which were not present in the included RCTs. This limitation is now explicitly addressed throughout the manuscript.

Importantly, the present review identifies a specific limitation in the literature, as most human studies have assessed inflammation-related biomarkers (e.g., IL-6 and TNF-α) rather than upstream regulators of senescence such as p16^INK4a^ or p21. The absence of eligible RCTs reporting these canonical markers highlights the need for future investigations that specifically target these endpoints.

Studies by Huang et al. [33], St. Sauver et al. [34], Falvino et al. [35], and Wong et al. [36] have provided further insight into the broader health implications of senescence and its biomarkers. Although these were not eligible for inclusion in our review, they reinforce the potential of lifestyle interventions, including physical activity, to influence immune function, inflammation, and biological ageing. Therefore, it is biologically plausible that exercise may influence the trajectory of age-related diseases by modulating senescence-related pathways, potentially contributing to improved health span. However, this hypothesis needs to be confirmed in future studies.

### Study limitations

Several limitations of this meta-analysis should be considered when interpreting the results. First, the small number of included studies and heterogeneity in exercise protocols make it difficult to generalize the findings. Research trials have employed a wide range of exercise modes (aerobic endurance, resistance training, high-intensity intervals, or mixed programs) with varying frequencies and durations (from weeks to years). Although this diversity reflects the many ways one can exercise, it complicates direct comparisons and meta-analyses. Senescence-related outcomes can differ depending on whether the program was, for instance, a 12-week walking regimen or a year-long resistance training program. Furthermore, the lack of standardization in measuring senescence biomarkers poses a methodological challenge and prevents direct comparisons with other studies. Additionally, most biomarkers are measured in the blood, which may not reflect intracellular or tissue-specific senescence mechanisms. Key regulators such as p16^INK4a^ and p21 require tissue or cell lysates, and their absence in the analyzed RCTs limits our ability to directly assess senescence modulation. Another factor to consider is the variability in the characteristics of the study population, including differences in age, health status, and baseline physical activity levels, which may have influenced the response to exercise. Notably, none of the included trials stratified participants based on the presence of chronic diseases or comorbidities despite their high prevalence in aging populations. This omission may have confounded biomarker responses, as conditions such as cardiovascular disease, type 2 diabetes, or systemic inflammation could independently modulate the SASP components. Future studies should incorporate clinical stratification or subgroup analyses to better understand how multimorbidity affects the relationship between exercise and cellular senescence. Finally, we acknowledge that our search strategy, while designed in collaboration with medical librarians and systematic review experts, focused on studies explicitly referencing “cellular senescence” or SASP-related terminology. As a result, some studies that assessed relevant inflammatory biomarkers without using senescence-specific descriptors may have been excluded. This decision was intended to ensure conceptual clarity and specificity aligned with the geroscience principles. We have clarified this rationale and acknowledged its potential limitations in the discussion section.

In addition, scientific literature highlights important challenges. For example, Sauver et al. [34] emphasized the variability in senescence biomarker expression across different populations, suggesting the need for standardized measurement protocols. Falvino et al. [35] highlighted the complex interplay between cellular aging and musculoskeletal health, raising questions regarding whether specific types of exercise yield different effects.

Finally, an important methodological consideration was the imbalance between the included studies in terms of sample size and quality. One of the studies analyzed (the LIFE Study) represents the largest and most rigorous trial conducted on exercise in older adults to date [32], whereas the second included trial was smaller in scale and less powered. This disproportion may introduce bias into the overall effect estimate and is especially relevant in meta-analyses with very few included studies. Consistent with these methodological constraints, the GRADE assessment classified the certainty of evidence as exceptionally low for all the outcomes evaluated. The results should be interpreted as inconclusive given the limited number of included trials and the broad confidence intervals observed in the meta-analyses.

### Research gaps and future directions

Unlike previous reviews that have broadly assessed inflammation in aging, our study specifically targeted senescence-related pathways, employing SASP components as proxies. Moreover, the absence of RCTs assessing upstream senescence markers highlights an overlooked, but important gap. Although the current evidence is insufficient to confirm that physical exercise modulates biomarkers of cellular senescence in older adults, our findings highlight the limited availability of high-quality randomized controlled trials exploring this relationship. The absence of significant effects in this meta-analysis underscores the need for more comprehensive studies in this field.

Future studies should incorporate a more comprehensive panel of senescence biomarkers, especially those underrepresented in the current literature, such as p16^INK4a^ and p21, to capture the multidimensional nature of cellular aging. Moreover, standardized protocols regarding intervention type, intensity, and duration are necessary to reduce heterogeneity and allow for more robust comparisons.

It would also be valuable to investigate the potential synergies between physical exercise and other emerging therapeutic approaches such as senolytics or nutritional strategies. As suggested by previous studies [33, 36], exploring multimodal interventions could provide insight into whether combining exercise with pharmacological or lifestyle therapies enhances the modulation of cellular senescence pathways.

Finally, future trials should consider including larger and more diverse populations to ensure generalizability and adopt designs that facilitate the exploration of causal mechanisms and long-term outcomes related to healthy aging and biological resilience. Future research should employ longitudinal designs to establish causality and long-term effects. Improving the certainty of evidence in this field will require coordinated efforts to generate larger high-quality trials that include upstream senescence markers and follow GRADE-aligned reporting practices.

## Conclusion

This systematic review and meta-analysis did not demonstrate a statistically significant effect of physical exercise on the biomarkers of cellular senescence in older adults. Although some studies included in this review investigated biomarkers such as SASP-related cytokines, the limited number of randomized controlled trials, the heterogeneity of interventions, and the variability in biomarker reporting restrict the interpretability of the findings. Importantly, SASP-related cytokines represent downstream inflammatory consequences of cellular senescence rather than its core molecular drivers and are therefore insufficient to fully define the primary senescence phenotype. Consequently, the absence of statistically significant effects should be interpreted with caution and not as evidence of a true lack of biological impact.

Importantly, no eligible RCTs have reported canonical markers of senescence such as p16^INK4a^ or p21, highlighting a critical gap in the literature. This absence underscores the need for future research using standardized protocols and targeting upstream regulators of senescence to better elucidate the role of physical activity in modulating cellular ageing. These regulators include canonical markers, such as p16^INK4a^ and p21, which provide more direct evidence of senescence pathways than cytokine-based SASP profiles, which may be influenced by multiple systemic and contextual factors.

Although substantial progress has been made in linking physical exercise to cellular senescence modulation, continued rigorous research is essential. Further well-designed, high-quality trials are required to determine whether exercise, either as a standalone intervention or in combination with other therapeutic strategies, can meaningfully influence cellular senescence and its associated clinical outcomes.

## Supplementary Information

Supplementary Material 1

## Data Availability

All data generated or analyzed during this study are included in this published article and its supplementary information files.

## Appendix A

### Full electronic search strategy

*PubMed Search String*.

(MeSH] OR “Exercise“[Mesh] OR “Physical Conditioning, Human“[Mesh] OR “Endurance Training“[Mesh] OR “Resistance Training“[Mesh] OR “exercise“[Title/Abstract] OR “physical activity“[Title/Abstract] OR “strength training“[Title/Abstract] OR “endurance training“[Title/Abstract])

AND.

[Title/Abstract (OR “Cellular Senescence“[Mesh] OR “Senescence“[Title/Abstract] OR “senescent cells“[Title/Abstract] OR “cell senescence“[Title/Abstract] OR “senescence markers“[Title/Abstract] OR “p16INK4a“[Title/Abstract] OR “β-galactosidase“[Title/Abstract] OR “senescence-associated secretory phenotype“[Title/Abstract] OR “SASP“[Title/Abstract])

AND.

(“Aged“[Mesh] OR “Aging“[Mesh] OR “Older adults“[Title/Abstract] OR “elderly“[Title/Abstract] OR “aged“[Title/Abstract] OR “aging“[Title/Abstract] OR “older people“[Title/Abstract])
