# Supplementary material for: Evidence gaps in the effects of exercise on SASP-Related biomarkers in older adults: a systematic review and meta-analysis of randomized controlled trials
Source: BMC Geriatr. 2026 Feb 6;26:322. doi: 10.1186/s12877-026-07025-5 (PMC12977871; doi:10.1186/s12877-026-07025-5)

**Supplementary Material:** Table 1

| **CCL2** | | | **IL6** | | | **IL8** | | | **TNF-**α | | |  |  |
| --- | --- | --- | --- | --- | --- | --- | --- | --- | --- | --- | --- | --- | --- |
| **Prior parameter** | **Prior distribution** | **95%CI amplitude** | **Hellinger distance** | **Signed informativeness** | **95%CI amplitude** | **Hellinger distance** | **Signed informativeness** | **95%CI amplitude** | **Hellinger distance** | **Signed informativeness** | **95%CI amplitude** | **Hellinger distance** | **Signed informativeness** |
| Effect (μ) | Berger-Deely | 14.439 | 0.258 | 0.049 | 1.505 | 0.482 | 0.041 | 3.687 | 0.523 | 0.031 | 2.074 | 0.391 | 0.002 |
|  | DuMouchel | 11.724 | 0.124 | 0.087 | 0.333 | 0.23 | 0.233 | 1.171 | 0.289 | 0.229 | 0.597 | 0.18 | 0.22 |
|  | Half-Cauchy | 10.262 | 0.057 | 0.153 | 1.917 | 0.531 | 0.111 | 2.814 | 0.496 | 0.038 | 2.043 | 0.406 | 0.034 |
|  | *Half-normal* | *** | *0* | *** | *** | *0* | *** | *** | *0* | *** | *** | *0* | *** |
|  | *Jeffreys* | *** | *0.21* | *** | *** | *0.444* | *** | *** | *0.494* | *** | *** | *0.39* | *** |
|  | Uniform | 12.385 | 0.16 | 0.052 | 5.971 | 0.641 | 0.289 | 7.101 | 0.635 | 0.207 | 6.152 | 0.554 | 0.222 |
| Heterogeneity (τ) | Berger-Deely | 99.339 | 1 | 0.216 | 2.28 | 0.979 | 0.075 | 5.301 | 0.994 | 0.062 | 3.052 | 0.988 | 0.003 |
|  | DuMouchel | 19.499 | 0.979 | 0.348 | 0.337 | 0.811 | 0.392 | 1.222 | 0.901 | 0.381 | 0.615 | 0.867 | 0.411 |
|  | Half-Cauchy | 8.103 | 0.97 | 0.577 | 2.2 | 0.932 | 0.182 | 2.995 | 0.961 | 0.109 | 2.307 | 0.938 | 0.138 |
|  | *Half-normal* | *** | *0* | *** | *** | *0* | *** | *** | *0* | *** | *** | *0* | *** |
|  | *Jeffreys* | *** | *1* | *** | *** | *0.972* | *** | *** | *0.992* | *** | *** | *0.987* | *** |
|  | Uniform | 9.427 | 0.987 | 0.426 | 7.931 | 0.947 | 0.364 | 8.477 | 0.974 | 0.306 | 8.031 | 0.953 | 0.31 |
| Prediction (Θ) | Berger-Deely | 127.834 | 0.591 | 0.148 | 2.664 | 0.569 | 0.045 | 7.069 | 0.606 | 0.035 | 3.735 | 0.493 | 0.002 |
|  | DuMouchel | 27.429 | 0.293 | 0.2 | 0.523 | 0.305 | 0.256 | 1.917 | 0.36 | 0.251 | 0.944 | 0.261 | 0.251 |
|  | Half-Cauchy | 13.944 | 0.153 | 0.336 | 3.392 | 0.605 | 0.116 | 5.024 | 0.578 | 0.048 | 3.613 | 0.498 | 0.042 |
|  | *Half-normal* | *** | *0* | *** | *** | *0* | *** | *** | *0* | *** | *** | *0* | *** |
|  | *Jeffreys* | *** | *0.474* | *** | *** | *0.529* | *** | *** | *0.576* | *** | *** | *0.491* | *** |
|  | Uniform | 25.736 | 0.356 | 0.215 | 12.852 | 0.698 | 0.296 | 15.776 | 0.701 | 0.22 | 13.315 | 0.623 | 0.23 |

Abbreviatures: CCL2: CC-chemokine ligand 2; IL6: Interleukin 6; IL8: Interleukin 8; TNFa: Tumor necrosis factor-α

Green: Posterior benchmark reference distributions.

**Supplementary Material:** Figure 1


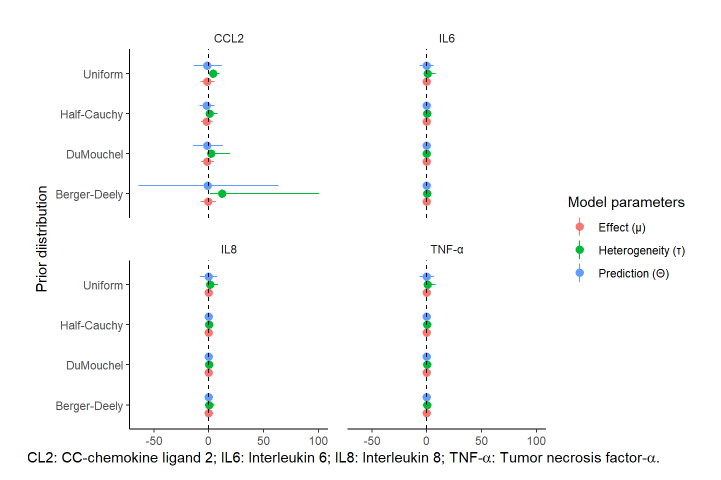


**Supplementary Material: Figure 2**


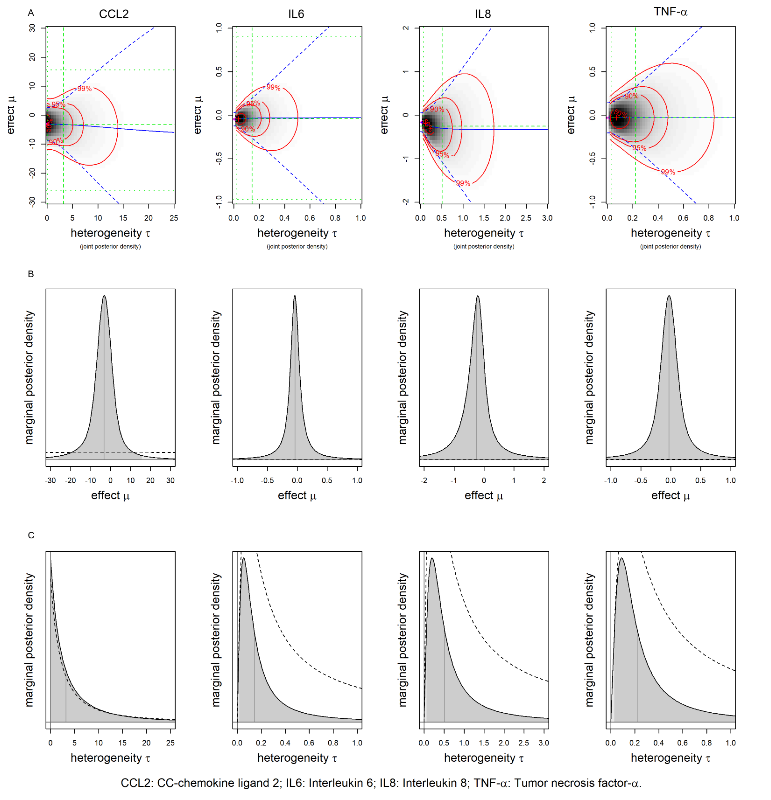


**Supplementary Material: Figure 3**


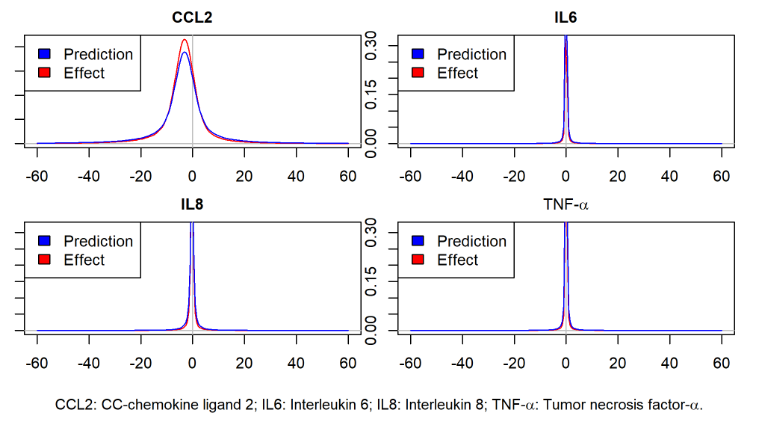

Supplement: Supplementary file 1 — Supplementary Material 1 [file 12877_2026_7025_MOESM1_ESM.docx]
